# Preoperative prediction of the pathological stage of advanced gastric cancer by ^18^F-fluoro-2-deoxyglucose positron emission tomography

**DOI:** 10.1038/s41598-022-14965-6

**Published:** 2022-07-05

**Authors:** Kota Yamada, Naoki Urakawa, Shingo Kanaji, Hiroshi Hasegawa, Masashi Yamamoto, Kimihiro Yamashita, Takeru Matsuda, Taro Oshikiri, Satoshi Suzuki, Yoshihiro Kakeji

**Affiliations:** Division of Gastrointestinal Surgery, Department of Surgery, Kobe University Graduate School of Medicine, 7-5-2 Kusunoki-chou, Chuo-ku, Kobe, 650-0017 Japan

**Keywords:** Gastric cancer, Cancer screening

## Abstract

In recent years, the usefulness of neoadjuvant chemotherapy for resectable advanced gastric cancer, particularly stage III, has been reported. Preoperative staging is mainly determined by computed tomography (CT), and the usefulness of ^18^F-fluoro-2-deoxyglucose positron emission tomography/CT (FDG-PET/CT) for gastric cancer has been limited in usefulness. The study aimed to evaluate the usefulness of FDG-PET/CT in preoperative diagnosis of advanced gastric cancer. We retrospectively enrolled 113 patients with gastric cancer who underwent preoperative FDG-PET/CT. All patients underwent gastrectomy with lymph-node dissection. The maximum standardized uptake value (SUVmax) of the primary tumor (T-SUVmax) and lymph nodes (N-SUVmax) were measured for all patients. The cutoff values of T-SUVmax for pathological T3/4 from receiver operating characteristic analysis were 8.28 for differentiated and 4.32 for undifferentiated types. The T-SUVmax and N-SUVmax cutoff values for pathological lymph-node metastasis were 4.32 and 1.82, respectively. Multivariate analysis showed that T-SUVmax for differentiated types was a significant predictor of pathological T3/4, and N-SUVmax was a significant predictor of lymph-node metastasis. In conclusion, the SUVmax of FDG-PET/CT was a useful predictor of pathological T3/4 and lymph-node metastasis in gastric cancer. The diagnosis by preoperative FDG-PET/CT is promising to contribute a more accurate staging of gastric cancer than by CT scan alone.

## Introduction

Gastric cancer (GC) is one of the most common malignancies and the third leading cause of cancer-related deaths worldwide^1^. Although diagnosis, surgical techniques, and adjuvant therapies for gastric cancer have advanced over the years, the prognosis for stage III and IV GC remains poor^2^. In recent years, neoadjuvant chemotherapy (NAC) has become the first choice for advanced GC due to the advantages of early chemotherapy for micro-metastasis, good general condition, and high treatment compliance in Western countries^3,4^. Similarly, a clinical study of NAC for GC with advanced lymph-node metastasis is currently underway by the Japan Clinical Oncology Group (JCOG)^5,6^. One of the problems of NAC therapy is the uncertainty of preoperative diagnosis of GC, which leads to unnecessary treatment and excessive invasion for early stage patients. Currently, the preoperative diagnosis of GC is mainly performed by esophagogastroduodenoscopy, endoscopic ultrasonography, and computed tomography (CT), but accuracy remains inadequate^7–9^. Thus, other useful predictors of advanced GC are essential for selection of an appropriate preoperative treatment.

^18^F-fluoro-2-deoxyglucose positron emission tomography/CT (FDG-PET/CT) is a valuable tool for diagnosis, staging, and identification of recurrence in many malignancies^10,11^. On the other hand, the role of preoperative FDG-PET/CT in GC has been limited in usefulness because of differences in FDG uptake by histological types. However, many studies have focused only on SUVmax of the primary tumor, few reports have evaluated the usefulness of preoperative FDG-PET/CT for the diagnosis of advanced GC using SUVmax cutoff values for the primary tumor and lymph nodes for histological type.

In this study, we measured the maximum standardized uptake values (SUVmax) of primary tumors and lymph nodes on FDG-PET/CT and evaluate the accuracy of FDG-PET/CT in diagnosing advanced GC, especially pT3.4, and pathological lymph node metastasis.

## Results

### Patients’ characteristics

The patients’ characteristics are shown in Table 1. Of 113 patients, pathologically, 60 patients (53.1%) were the differentiated and 53 (46.9%) were the undifferentiated type. Fifty-nine (52.2%) patients were clinically diagnosed as T3/4 by esophagogastroduodenoscopy and CT. Fifty-eight (51.3%) patients were clinically diagnosed as positive for lymph-node metastasis on the basis of the lymph-node diameters measured by CT. Pathological T3/4 was confirmed in 56 (49.6%) patients and lymph-node metastasis in 54 (47.8%) patients.

**Table 1:** Patient characteristics and surgical procedure.

	Value (n = 113)
Age, years, median (range)	73 (37–90)
**Sex**	
Male	77
Female	36
**Tumor location**	
Upper	26
Middle	54
Lower	33
**Histological type**	
Papillary	1
Well- and moderately differentiated	59
Poorly differentiated	43
Signet ring cell	8
Mucinous	2
**cT status**^**a**^	
T2	54
T3	47
T4	12
**cN status**^**a**^	
N0	55
N1	42
N2	14
N3	2
**cStage**^**a**^	
I	36
II	40
III	34
IV	3
**pT status**^**a**^	
T1	42
T2	15
T3	28
T4	28
**pN status**^**a**^	
N0	59
N1	21
N2	11
N3	22
**pStage**^**a**^	
I	51
II	26
III	28
IV	8
**Gastrectomy**	
Distal gastrectomy	71
Total gastrectomy	42
**Lymph-node dissection**	
D1+	34
D2	79
Number of lymph node dissections, median (range)	39 (11–97)

^a^Classified based on TNM staging according to the Japanese Classification of Gastric Carcinoma, 3rd English edition^21^.

### Relationship between histological type and SUVmax

The median T-SUVmax and N-SUVmax were 5.08 (range, 0.78–34.1) and 1.57 (range, 0.74–14.3), respectively. T-SUVmax (median, range) was not significantly different between each tumor location (Upper: 5.00, 2.00–25.23; Middle: 3.62, 0.78–21.55; Lower: 6.90, 1.43–34.05, *P* = 0.14). There was also no significant difference between type1.2 (4.98, 0.78–25.23) and type3.4 (4.50, 2.00–34.05) in macroscopic type (*P* = 0.65). In pathological depth of tumor, T-SUVmax (median, range) was significantly higher in T3, 4 (7.33, 2.00–34.05) than T1, 2 (3.13, 0.78–13.02) (*P* < 0.005). We evaluated the relationship between histological type and SUVmax. The median T-SUVmax was 6.34 (1.18–34.1) for the differentiated type and 3.57 (0.78–21.6) for the undifferentiated type. The median N-SUVmax was 1.52 (0.74–12.3) for the differentiated type and 1.67 (0.89–14.3) for the undifferentiated type. The T-SUVmax were higher in the differentiated type than in the undifferentiated type (*P* = 0.02), but there was no significant difference in the N-SUVmax (*P* = 0.21).

### Cutoff SUVmax for diagnosis of pathological T3/4 tumor and lymph-node metastasis

**Figure 1:**
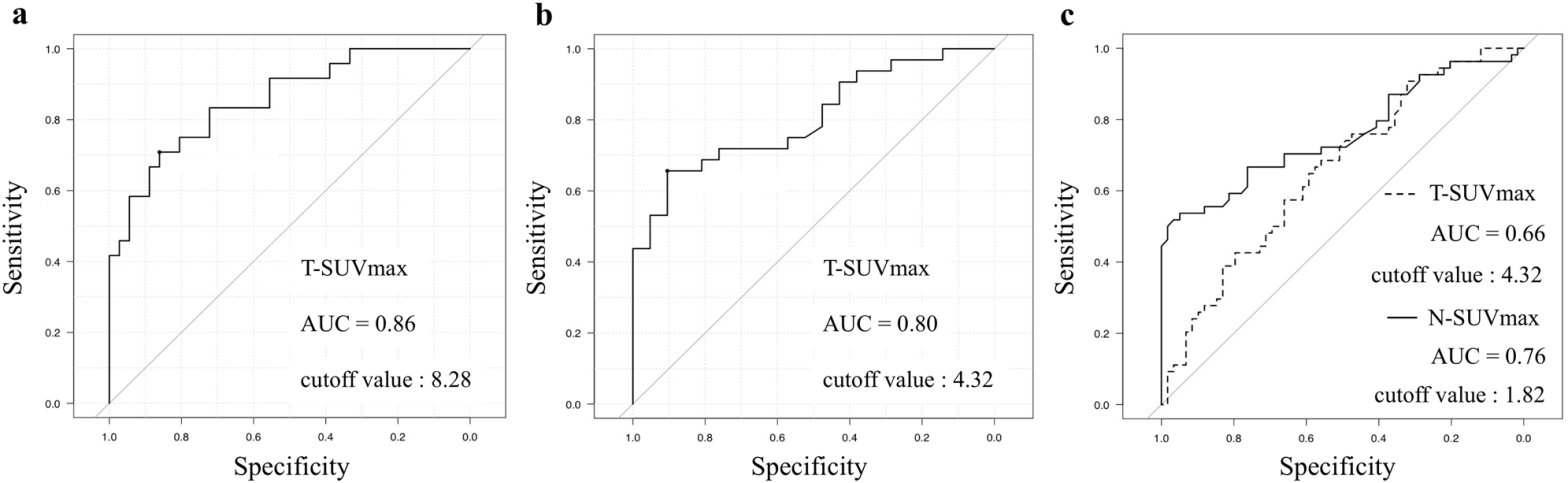
(**a,b**) Receiver operating characteristic (ROC) curve for assessment of the confidence of T-SUVmax to predict pathological T3 or T4 tumor. (**a** differentiated type, **b** undifferentiated type). **c** ROC curve for assessment of the confidence of T-SUVmax and N-SUVmax to predict pathological lymph-node metastasis.

The AUCs of the T-SUVmax values to predict pT3/4 determined by ROC curve analysis were 0.86 (95% CI, 0.76–0.95) for the differentiated type and 0.80 (0.69–0.92) for the undifferentiated type (Fig. 1a,b). The AUCs of the T-SUVmax and N-SUVmax for predicting pathological lymph-node metastasis were 0.66 (0.56–0.76) and 0.76 (0.67–0.85), respectively (Fig. 1c). The cutoff values of T-SUVmax for pT3/4 were 8.28 for the differentiated type and 4.32 for the undifferentiated type, and the cutoff values of T-SUVmax and N-SUVmax for pathological lymph-node metastasis were 4.32 and 1.82, respectively.

### Usefulness of T-SUVmax for diagnosis of the tumor invasion depth

The results of the univariate and multivariate logistic regression analysis for pT3/4 are shown in Table 2. In the differentiated type, univariate logistic regression showed that high preoperative CA19-9, cT3/4, cN positive, and high T-SUVmax (≥ 8.28) were significantly associated with pT3/4. In the multivariate analysis, cT3/4 (Odds ratio [OR], 62.1; 95% CI, 5.32–724.0; *P* < 0.01), high T-SUVmax (OR, 8.88; 95% CI, 1.35–58.3; *P* = 0.02) were significantly predictive factors for pT3/4, but in the undifferentiated type, cT3/4 and high T-SUVmax (≥ 4.32) were not.

**Table 2:** Univariate and multivariate analysis for pathological T3/T4 tumors.

	Differentiated type				Undifferentiated type			
	Univariate analysis		Multivariate analysis		Univariate analysis		Multivariate analysis	
	OR (95% CI)	*P* value	OR (95% CI)	*P* value	OR (95% CI)	*P* value	OR (95% CI)	*P* value
**Age (years)**								
≥ 75/ < 75	1.98 (0.61–6.56)	0.28			1.54 (0.43–5.83)	0.57		
**Sex**								
Male/female	3.43 (0.78–21.5)	0.08			0.90 (0.25–3.18)	1.00		
**Pre-CEA (ng/ml)**								
≥ 5.0/ < 5.0	1.73 (0.47–6.50)	0.38			4.21 (0.76–44.3)	0.09		
**Pre-CA19-9 (U/ml)**								
≥ 37.0/ < 37.0	5.53 (1.32–28.5)	0.01*	3.72 (0.51–27.3)	0.20	2.16 (0.34–24.2)	0.46		
**cT status**								
T3–4/T2	85.2 (10.7–3941.9)	< 0.01**	62.1 (5.32–724.0)	< 0.01**	17.1 (3.21–177.4)	< 0.01**	5.37 (0.99–29.10)	0.05
**cN status**								
Positive/negative	5.04 (1.49–18.6)	< 0.01**	2.50 (0.41–15.4)	0.32	1.59 (0.46–5.62)	0.57		
**T-SUVmax**								
High/low	14.2 (3.58–68.1)	< 0.01**	8.88 (1.35–58.3)	0.02*	14.2 (3.29–78.3)	< 0.01**	5.91 (0.82–42.80)	0.08

*OR* odds ratio, *CI*  confidence interval, *pre* preoperative, *SUV max*  maximum standardized uptake value.

*P value < 0.05, **P value < 0.01.

### Usefulness of N-SUVmax for diagnosis of lymph-node metastasis

Univariate logistic regression revealed that Male, cT3/4, cN positive, high T-SUVmax (≥ 4.32), and high N-SUVmax (≥ 1.82) were significantly associated with lymph-node metastasis. In the multivariate analysis, Male (OR, 4.36; 95% CI, 1.43–13.3; *P* < 0.01), cT3/4 (OR, 4.10; 95% CI, 1.31–12.9; *P* = 0.02), and high N-SUVmax (OR, 16.5; 95% CI, 3.84–70.5; *P* < 0.01) were significant predictors of lymph-node metastasis (Table 3). And high N-SUVmax was also a significant predictor of lymph node metastasis in multivariate analysis of differentiated (OR, 9.32; 95% CI, 1.34–64.8; *P* = 0.02) and undifferentiated (OR, 15.1; 95% CI, 2.42–93.5; *P* < 0.01) respectively. To evaluate the diagnostic ability of CT and FDG-PET/CT, we compared their sensitivity, specificity, positive predictive value (PPV), negative predictive value (NPV), and accuracy (Table 4). Although the sensitivity, specificity, PPV, and NPV for diagnosing lymph-node metastasis based on lymph-node diameters by CT were 68.5%, 69.5%, 67.3%, and 70.7%, respectively, the sensitivity, specificity, PPV, and NPV for diagnosing lymph-node metastasis based on N-SUVmax by FDG-PET/CT were 53.7%, 94.9%, 90.6%, and 69.1%, respectively. The accuracy of diagnosis of lymph-node metastasis was higher by FDG-PET/CT than by CT (75.2% vs. 69.0%).

**Table 3:** Univariate and multivariate analysis for pathological lymph-node metastasis.

	Univariate analysis		Multivariate analysis	
	OR (95% CI)	*P* value	OR (95% CI)	*P* value
**Age (years)**				
≥ 75/ < 75	1.44 (0.64–3.31)	0.44		
**Sex**				
Male/female	3.43 (1.37–9.14)	< 0.01**	4.36 (1.43–13.3)	< 0.01**
**Histology**				
Undifferentiated/differentiated	1.46 (0.65–3.29)	0.35		
**Pre-CEA (ng/ml)**				
≥ 5.0/ < 5.0	0.93 (0.36–2.39)	1.00		
**Pre-CA19-9 (U/ml)**				
≥ 37.0/ < 37.0	1.75 (0.62–5.15)	0.34		
**cT status**				
T3–4/T2	4.24 (1.82–10.26)	< 0.01**	4.10 (1.31–12.9)	0.02*
**cN status**				
Positive/negative	4.88 (2.08–11.9)	< 0.01**	2.03 (0.75–5.50)	0.16
**T-SUV max**				
≥ 4.32/ < 4.32	2.74 (1.20–6.4)	0.01*	0.58 (0.18–1.85)	0.36
**N-SUV max**				
≥ 1.82/ < 1.82	21.0 (5.73–118.2)	< 0.01**	16.5 (3.84–70.5)	< 0.01**

*OR* odds ratio, *CI* confidence interval, *pre* preoperative, *SUVmax*  maximum standardized uptake value.

*P value < 0.05, **P value < 0.01.

**Table 4:** Diagnostic ability of CT scan and FDG-PET/CT to detect pathological lymph-node metastasis.

	Sensitivity (%)	Specificity (%)	Positive predictive value (%)	Negative predictive value (%)	Accuracy (%)
CT scan (lymph-node diameter ≥ 8 mm)	68.5	69.5	67.3	70.7	69.0
FDG-PET/CT (N-SUVmax ≥ 1.82)	53.7	94.9	90.6	69.1	75.2
CT scan and FDG-PET/CT^a^	50.0	98.3	96.4	68.2	75.2

*SUV max* maximum standardized uptake value.

^a^When both images were positive, the result was considered positive.

## Discussion

The SUVmax in primary GC tumors has been shown to correlate with tumor invasion depth^12–14^, but few studies have predicted pT status by using a cutoff value. By analyzing the relationship between histological type and SUVmax, we found that the uptake of FDG in primary tumors of GC depended on histological type, with higher uptake in differentiated types and lower uptake in undifferentiated types. Therefore, in undifferentiated types, multivariate analysis did not show that the T-SUVmax was useful for diagnosis of pT3/4. This finding is consistent with those of several previous reports and explains the low utility of FDG-PET/CT in GC^15–17^. However, our results showed that the T-SUVmax was a significant predictor of pT3/4 diagnosis in differentiated types by using a cutoff value with the ROC curve.

In the diagnosis of lymph-node metastasis, Oh et al. have shown that T-SUVmax on FDG-PET/CT is a useful predictor of pathological lymph-node metastasis in GC^18^. These studies focused only on the metabolic activity of the primary tumor. We considered that using the metabolic activity of lymph nodes as a predictor of lymph-node metastasis would further improve the diagnostic accuracy. Tsunoda et al. revealed that in rectal cancer, a high N-SUVmax (> 1.5) was more useful in preoperative diagnosis of lymph-node metastasis than was the lymph-node diameter^19^. Our study showed similar results in GC, and N-SUVmax was a useful predictor for diagnosing pathological lymph-node metastasis regardless of histological type.

Fukagawa et al. reported that it was difficult to accurately diagnose pathological lymph-node metastasis and reported the sensitivity and specificity of CT without FDG-PET/CT were 62.5% and 65.7%, respectively^20^. In our analysis, the accuracy of CT alone in diagnosing pathological lymph-node metastasis was similar to their results, but the combination of diagnosis by FDG-PET/CT improved the accuracy of preoperative diagnosis of pathological lymph-node metastasis. Even in cases diagnosed as positive for lymph-node metastasis by CT, if N-SUVmax was low in FDG-PET/CT, 94% (17/18) of the cases were negative for pathological lymph-node metastasis, which is also useful for diagnosis of negative prediction.

Considering the cost of FDG-PET/CT, it may not be practical to perform it preoperatively for all patients with GC. Therefore, it is important to select patients with comorbidities who are expected to experience more adverse events from NAC treatment, and patients with poor renal function who cannot undergo contrast-enhanced CT. The use of FDG-PET/CT combined with conventional diagnostic methods, such as esophagogastroduodenoscopy and CT, can avoid unnecessary NAC treatment for such patients and contribute to appropriate treatment selection.

This study had several limitations. First, this was a single-institution retrospective study, so selection bias was possible. However, FDG-PET/CT is expensive, and it is valuable to demonstrate the significance of this study in a small number of cases. In addition, because it was performed according to our institution's protocol in general, special pretreatment, such as drinking milk or water to improve the evaluation of GC by FDG-PET/CT, was not performed.

Second, the SUV of small-sized primary tumors and lymph nodes could have been underestimated due to partial volume effects.

Third, in this study, we used SUVmax, which can be easily measured and can also be used to evaluate lymph nodes where volumetric parameters are not available. However, metabolic tumor volume parameters in the evaluation of the primary tumor may have been more useful.

In conclusion, preoperative FDG-PET/CT staging of advanced GC is promising to contribute to the selection of appropriate treatment strategies. Future prospective research is needed to determine how the improved diagnostic accuracy gained by adding FDG-PET/CT to conventional CT scans can lead to improved treatment outcomes.

## Patients and methods

### Patients

From January 2014 to December 2019, 113 patients who had undergone gastrectomy for adenocarcinoma of GC at Kobe University Hospital were analyzed retrospectively in this study. These patients had been diagnosed with gastric adenocarcinoma by esophagogastroduodenoscopy and had undergone preoperative CT and FDG-PET/CT for initial staging. Preoperative diagnoses of the depth of tumor invasion (T status) and lymph-node metastasis (N status) were diagnosed by esophagogastroduodenoscopy and CT. For evaluation of N status, a size ≥ 8 mm measured by CT was considered positive with reference to the JCOG 1302A study^20^. All patients received distal or total gastrectomy with D1 + or D2 lymph-node dissection. All lymph nodes suspected to be metastatic on preoperative CT or FDG-PET/CT were dissected in surgery. Patients who received any preoperative treatments, such as chemotherapy or radiotherapy, were excluded. The analysis for statistics was divided by 75 years of age, which is the target age group for most of the JCOG clinical trials for NAC in advanced GC. The diagnosis had been confirmed by histopathological examination according to the Japanese Classification of Gastric Carcinoma, 3rd English edition^21^. Pathologically, papillary and well- and moderately differentiated adenocarcinomas are defined as differentiated, whereas poorly differentiated, mucinous, and signet-ring-cell adenocarcinomas are defined as undifferentiated. This study was approved by the Ethics Committee of Kobe University (No. B210017).

### FDG-PET/CT protocol

**Figure 2:**
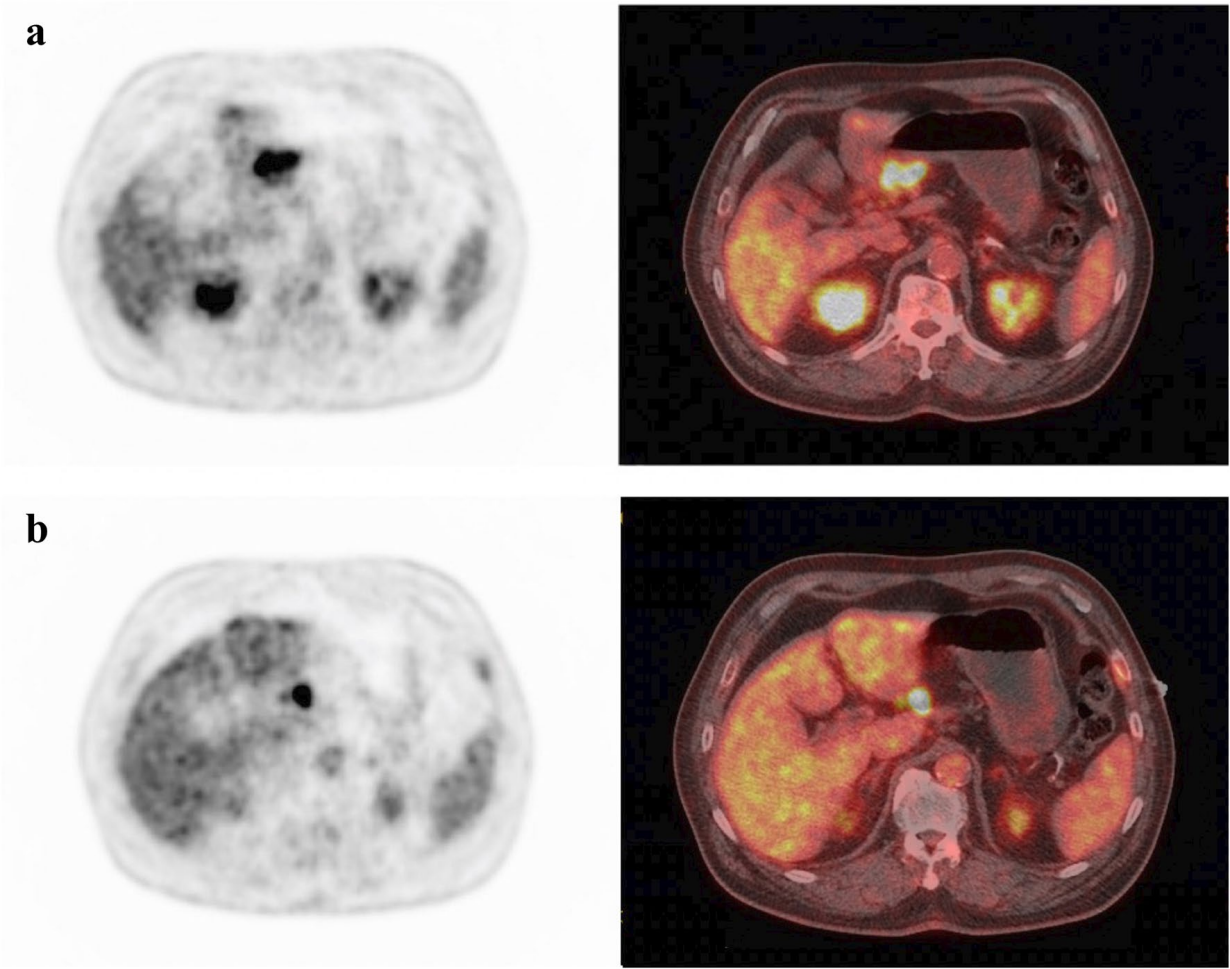
Preoperative FDG-PET/CT of a 72-year-old man showing FDG uptake (**a** T-SUVmax, 11.53; **b** N-SUVmax, 12.29). This patient underwent subtotal gastrectomy and stage III gastric cancer was confirmed (pT3N2M0 pStageIIIA, well-differentiated tubular adenocarcinoma).

Whole-body FDG-PET scans were performed on a PET scanner (Philips Allegro, Philips Medical System, Best, the Netherlands). All patients fasted for ≥ 6 h before injection of 222–333 MBq (6–9 mCi) of FDG to minimize the effects of gastric filling and had blood glucose levels of < 160 mg/dl at the time of injection. Approximately 1 h after IV administration of 222 to 333 MBq (6–9 mCi) of FDG, a static emission scan was performed. Emission PET scans were reconstructed by using the row-action maximum-likelihood algorithm resulting in a 128 × 128 matrix. After the PET scans, the patients underwent CT scans performed at 120 kV and 80 mA. All PET and CT images were integrated by using automatic image-fusion software (Syntegra; SUN Microsystems). The T-SUVmax and N-SUVmax for all patients were measured by FDG-PET/CT (Fig. 2). Two surgeons analyzed all PET images separately under the supervision of one nuclear medicine physicians. When discrepancies were detected, interpretations were achieved via consensus. Pathological results were blinded to the analyst.

In detectable lesions, SUVmax was measured from each lesion. In the non-detectable cases, SUVmax was measured from the corresponding site identified by esophagogastroduodenoscopy or enhanced CT scans.

### Statistical analyses

Statistical analysis was performed to determine the relationships between clinicopathological parameters, including the SUVmax, and pathological T3/4 (pT3/4) tumor and lymph-node metastasis by using the Mann–Whitney *U*-test for continuous variables and Fisher’s exact test for categorical variables, as appropriate. All *P* values < 0.05 were considered to be indicative of statistical significance. A multiple logistic regression model was used to identify useful predictors for diagnosis of pT3/T4 tumors and lymph-node metastasis. Covariates found to be significant in the univariate analysis at *P* < 0.05 were included in the multivariate model. Receiver operating characteristic (ROC) analysis was performed to assess the confidence of the SUVmax for predicting pT3/T4 tumors and lymph-node metastasis, and the areas under the curves (AUCs) were measured. The optimal cutoff value was determined by ROC analysis. All statistical analyses were performed with EZR^22^ (Saitama Medical Center, Jichi Medical University, Saitama, Japan), which is a graphical user interface for R (The R Foundation for Statistical Computing, Vienna, Austria). More precisely, it is a modified version of R commander designed to add statistical functions frequently used in biostatistics.

### Human rights statement and informed consent

All procedures followed were in accordance with the ethical standards of the responsible committee on human experimentation (institutional and national) and with the Helsinki declaration of 1964 and later versions. Informed consent to be included in the study, or the equivalent, was obtained from all patients.

## Data Availability

The datasets generated and/or analyzed during the current study are available from the corresponding author on reasonable request.
